# Lactate dynamics in paediatric patients with severe sepsis: insights from a prospective cohort study

**DOI:** 10.1186/s12887-024-04809-9

**Published:** 2024-05-18

**Authors:** Tarek A. Abdelaziz, Nehad Ahmed Karam, Weaam Ibrahim Ismail, Nahed Mohamed Ali Askary, Eman Gamal Baz

**Affiliations:** 1 Paediatric Department, Faculty of Medicine, Zagazig University, Zagazig city, Egypt; 2 Clinical Pathology Department, Faculty of Medicine, Zagazig University, Zagazig city, Egypt

**Keywords:** Clearance, Delta-lactate, Kinetics, Lactate, Lac H0, Lac H6, Mortality, Sepsis, Shock

## Abstract

**Background:**

Sepsis is an infection-related systemic inflammatory response that often leads to elevated lactate levels. Monitoring lactate levels during severe sepsis is vital for influencing clinical outcomes. The aim of this study was to assess the association between plasma lactate levels and mortality in children with severe sepsis or septic shock.

**Methods:**

The current prospective study was conducted in the PICU of University Children’s Hospital. The International Paediatric Sepsis Consensus Conference criteria for Definitions of Sepsis and Organ Failure in 2005 were used to diagnose patients with sepsis. We measured plasma lactate levels upon admission (Lac H0) and 6 h later (Lac H6). The static indices included the absolute lactate values (Lac H0 and Lac H6), while the dynamic indices included the delta-lactate level (ΔLac) and the 6-hour lactate clearance. The 6-hour lactate clearance was calculated using the following formula: [(Lac H0–Lac H6)100/Lac H0]. ΔLac was calculated as the difference between the Lac H0 and Lac H6 levels. Patient survival or death after a PICU stay was the primary outcome.

**Results:**

A total of 46 patients were included in this study: 25 had septic shock, and 21 had severe sepsis. The mortality rate was 54.3%. The Lac H0 did not significantly differ between survivors and nonsurvivors. In contrast, the survivors had significantly lower Lac H6 levels, higher ΔLac levels, and higher 6-hour lactate clearance rates than nonsurvivors. Lactate clearance rates below 10%, 20%, and 30% were significantly associated with mortality. The best cut-off values for the lactate clearance rate and Lac H6 for the prediction of mortality in the PICU were < 10% and ≥ 4 mmol/L, respectively. Patients with higher Lac H6 levels and lower lactate clearance rates had significantly higher PICU mortality based on Kaplan–Meier survival curve analysis.

**Conclusions:**

This study highlights the significance of lactate level trends over time for the prediction of mortality in the PICU in patients with severe sepsis or septic shock. Elevated lactate levels and decreased lactate clearance six hours after hospitalisation are associated with a higher mortality rate.

## Introduction

Severe sepsis and septic shock in pediatric patients can cause an inappropriate immunological response, which can lead to tissue damage, organ failure, and death during their stay in the pediatric intensive care unit (PICU). Septic shock is one of the most serious medical emergencies, causing high lactate levels. Even in high-income countries, it contributes to a 10% mortality rate [1–3].

Lactate produced in the human body is excreted through the liver and kidneys. Hyperlactatemia may be caused by decreased clearance, excessive production, or both. Elevated lactate levels occur in patients with shock, renal or hepatic failure, diabetic ketoacidosis, some inborn errors of metabolism, severe hypoxemia, and cardiac arrest. Monitoring lactate levels during severe sepsis and septic shock is vital for influencing clinical outcomes [2, 3].

Critically ill patients may remain in compensated septic shock with normal blood pressure and urine output. Therefore, relying solely on maintaining normal physiological parameters such as vital signs and urinary output may be insufficient in these patients, as prompt detection and treatment of inadequate blood supply are crucial for their treatment. The plasma lactate value serves as one of the biological variables evaluated, and it can be employed to identify hypoperfusion and assess the effectiveness of resuscitation [4, 5].

According to previous studies, single lactate measurements are not consistently correlated with death and have been used as markers of hypoperfusion; however, their sensitivities and specificities are unclear. Additionally, lactate clearance is an approximate percentage of the reduction in lactate levels following early resuscitation and is a plausible method for predicting mortality [6]. Increased lactate levels correlate with poor outcomes, and survival can be predicted in critically ill adults with septic shock through early lactate clearance [7–10]. However, the predictive accuracy of lactate clearance in children with severe sepsis has not been fully investigated [4, 11]. Additionally, there are inadequate data available for predicting mortality during hospitalisation using lactate measurements. Therefore, this study aimed to assess the prognostic value of plasma lactate levels in children with severe sepsis or septic shock.

## Subjects and methods

### Patient inclusion/exclusion criteria

The present prospective observational study was conducted between November 2021 and June 2022. We consecutively recruited all patients aged 28 days to 16 years with severe sepsis or septic shock from the PICU of the University Children’s Hospital. The International Paediatric Sepsis Consensus Conference criteria for Definitions of Sepsis and Organ Failure in 2005 were used to diagnose sepsis [12]. Severe sepsis was defined as one of the following: cardiovascular organ dysfunction, acute respiratory distress syndrome, or dysfunction of two or more additional organs. Septic shock includes sepsis and cardiovascular organ dysfunction [12]. The 2020 Surviving Sepsis Campaign Guidelines have been used for treatment recommendations [13]. Using OPEN-EPI, the estimated sample size at 80% power and 95% confidence intervals was 46 patients, assuming that the mean lactate levels at admission were 3.3 ± 1.7 for survivors and 5.4 ± 3.1 for nonsurvivors. We excluded patients who received treatment at a different healthcare facility, had other causes of shock unrelated to sepsis, had malignant diseases, were receiving immunosuppressive treatment, or had conditions known to cause elevated lactate levels, such as inborn metabolic errors. The therapeutic endpoints targeted during fluid resuscitation include the following: first, a normalised systolic blood pressure; second, strong distal pulses comparable to central pulses; third, appropriate skin perfusion; and fourth, a urine amount equal to or greater than 1 mL/kg/h [14].

### Patient workup

The clinical data of the recruited patients were collected and subjected to further investigation. Complete blood count, electrolyte levels, coagulation test results, liver and kidney function, and serum calcium, phosphorus, magnesium, and C-reactive protein levels were assessed according to the manufacturer’s guidelines. Blood cultures for each patient were obtained, and cultures from different sites, including tracheal aspirate, cerebrospinal fluid (CSF), urine, and central venous catheter (CVC) analyses, were obtained only when clinically indicated. Cultures from the blood samples were incubated in a BACT/ALERT3D system (Biomerieux, France), whereas other cultures were incubated on plates containing suitable media. A VITEK2 Compact device (Biomerieux, France) was used to identify and test for antibiotic susceptibility in the positive cultures.

### Lactate kinetic measurements

We measured plasma lactate levels upon admission (Lac H0), within the first hour of PICU admission, and after 6 h (Lac H6). The static indices included the absolute lactate values (Lac H0 and Lac H6), while the dynamic indices included the lactate clearance and delta-lactate (ΔLac) after 6 h. ΔLac was identified as the alteration in lactate level after 6 h and was calculated as the difference between the Lac H0 and Lac H6 levels. Following the initial resuscitation, the 6-hour lactate clearance was the estimated percentage drop in lactate plasma levels and was calculated as [(Lac H0-Lac H6) 100/Lac H0] [11]. Blood specimens were collected aseptically from large veins using a tourniquet in suitable tubes or collection containers supplemented with Na-fluoride/K-oxalate or Na-fluoride/Na-heparin plasma and centrifuged. Plasma levels of lactate were measured using spectrophotometry on a 6000 Roche Cobas (c501) (Roche Diagnostics, Switzerland). Lactate oxidase transforms lactate into pyruvate. Peroxidase was used to create a colored dye from the hydrogen peroxide produced in the initial reaction. The L-lactate concentration was proportional to the intensity of the color.

### The primary outcome

The primary outcome was patient survival or death after a PICU stay. We divided our subjects into two distinct groups: survivors and nonsurvivors.

### Statistical analysis

We performed the data analysis using SPSS version 26 (Statistical, Armonk, NY, USA). The chi-square (X2) test was employed for comparisons of frequencies and percentages, which are categorical variables. The means and standard deviations (SDs) or medians and interquartile ranges (IQRs) of the quantitative variables were used to describe the data. We checked assumptions for parametric tests using the Kolmogorov‒Smirnov and Shapiro‒Wilk tests. We compared quantitative data across the two distinct groups using the independent sample t test (for normally distributed data) and the Mann‒Whitney test (for nonnormally distributed data). The associations between various lactate metrics and hospital mortality were examined through a logistic regression model. The multivariate model was adjusted for age, gender, and comorbidities. These confounders have the potential to influence both lactate metrics and hospital mortality. By including these factors as covariates in our analysis, we aimed to control for their potential impact and isolate the specific association between lactate metrics and mortality. Kaplan–Meier survival curve analysis was used. The results are expressed as odds ratios with 95% confidence intervals. We set the statistical significance at *P* < 0.05.

## Results

**Fig. 1:**
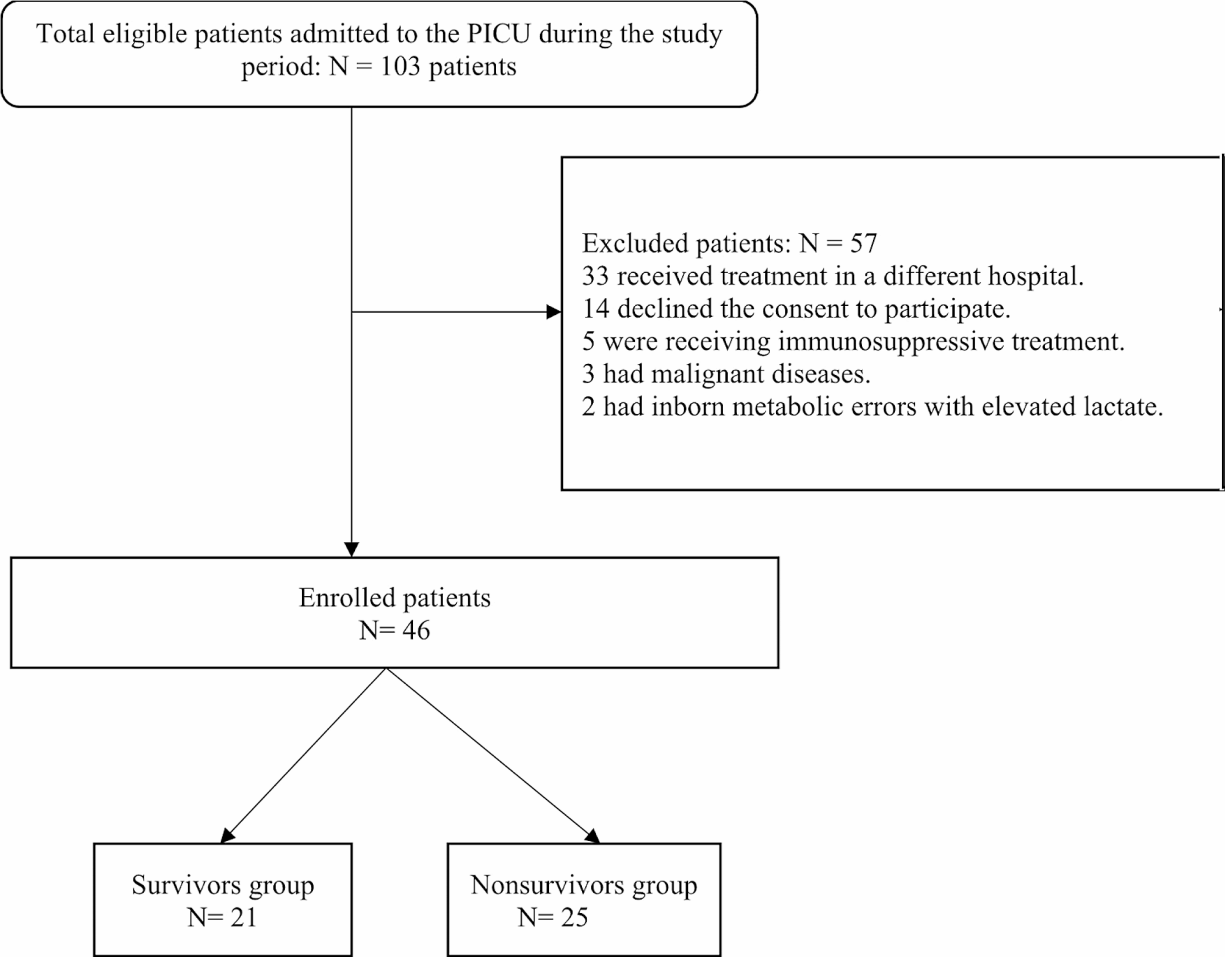
Flowchart of the study population

Figure 1 displays the flowchart of the research population. In total, 46 patients were enrolled in the study. There were 21 and 25 patients in the survivor and nonsurvivor groups, respectively. Age, sex, length of PICU stay, diagnosis, and duration of mechanical ventilation did not significantly differ between the two distinct groups **(**Table 1**)**. *Klebsiella pneumonia, Escherichia coli, Staphylococcus aureus, and Acinetobacter baumannii* were detected in 38%, 24%, 10%, and 5% of the survivors, respectively, whereas they were detected in 24%, 16%, 16%, and 16%, respectively, of the nonsurvivors.

**Table 1:** Basic characteristics of the study cohort

	Overall (*N* = 46)	Survivors (*N* = 21)	Nonsurvivors (*N* = 25)	Test of significance	*P value
Age (months) median (IQR)	11 (2–165)	12 (2–165)	8 (2–144)	U = 213	0.27
Sex					
Male Female	26 (57%) 20 (43%)	14 (66.7%) 7 (33.3%)	12 (48%) 13 (52%)	χ2 = 1.62	0.20
**Sepsis Grade**					
Severe sepsis	21 (46%)	11 (52.4%)	10 (40%)	χ2 = 0.71	*0.01
Septic shock	25 (54%)	10 (47.6%)	15 (60%)		
MV duration (days) median (IQR)	14.5 (1–33)	14 (3–33)	15 (1–33)	U = 239	0.61
PICU staying (days) median (IQR)	16 (1–36)	18 (5–36)	15 (1–33)	U = 186	0.09
**Diagnosis**					
Pneumonia	16 (35%)	8 (38.1%)	8 (32%)	χ2 = 8.67	0.73
Complicated bronchiolitis	1 (2%)	0 (0%)	1 (4%)		
Severe stridor	1 (2%)	1 (4.8%)	0 (0%)		
Pneumothorax secondary to pneumonia	1 (2%)	1 (4.8%)	0 (0%)		
Aspiration pneumonia	2 (4%)	1 (4.8%)	1 (4%)		
Status epilepticus	6 (13%)	3 (14.3%)	3 (12%)		
Cerebral sinovenous thrombosis	1 (2%)	1 (4.8%)	0 (0%)		
Meningoencephalitis	2 (4%)	1 (4.8%)	1 (4%)		
Aplastic anemia	1 (2%)	1 (4.8%)	0 (0%)		
Complicated gastroenteritis	6 (13%)	2 (9.5%)	4 (16%)		
Heart failure	7 (15%)	2 (9.5%)	5 (20%)		
Superior mediastinal syndrome	1 (2%)	0 (0%)	1 (4%)		
Infective endocarditis	1 (2%)	0 (0%)	1 (4%)		
WBCs (x103/mm3), median (IQR)	24.5 (7–48)	24 (14–48)	25 (7–38)	U = 253.5	0.84
Hemoglobin (gm/dL), median (IQR)	9.85 (5.7–13.8)	9.3 (5.7–11.4)	9.9 (7–13.8)	U = 199	0.16
Platelets (x103/mm3), median (IQR)	318 (16–637)	250 (16– 547)	355 (25–637)	U = 195	0.14
Blood urea nitrogen (mg/dL), median (IQR)	16.35 (6.2 − 67.7)	22 (6.3–67.7)	15.1 (6.2–58)	U = 238	0.59
Creatinine (mg/dL), median (IQR)	0.45 (0.15–4.8)	0.5 (0.2–4.8)	0.3 (0.15–3.8)	U = 193.5	0.13
Sodium (mmol/L), median (IQR)	135 (114–152)	136 (117 − 148)	134 (114–152)	U = 224	0.39
Potassium (mmol/L), median (IQR)	3.95 (2.1–7)	4 (2.2–7)	3.9 (2.1–5.9)	U = 252	0.83
Calcium (mg/dL), median (IQR)	9 (6.5–12.80)	8.9 (7.9–11.2)	9 (6.5–12.8)	U = 245	0.69
Magnesium (mg/dL), median (IQR)	2.21 (0.9–3.1)	2.1 (1.5–2.68)	2.3 (0.9–3.1)	U = 177.5	0.06
Phosphorus (mg/dL), median (IQR)	4.2 (2.2–8)	4 (2.2–6.17)	4.5 (2.6–8)	U = 191	0.12
Serum protein					
C-reactive protein (mg/L), median (IQR)	140.5 (53–445)	130 (40–215)	155 (53–445)	*U = 212	*0.04
Total Protein (gm/dL), median (IQR)	5.6 (3–7.6)	5.5 (3.2–7.5)	5.6 (3–7.6)	U = 234	0.53
Albumin (gm/dL), median (IQR)	3.37 (2–5.3)	3.34 (2.3–4)	3.4 (2–5.3)	U = 246	0.72

IQR; interquartile quartile range, Mann‒Whitney U test; MV, mechanical ventilation; PICU, paediatric intensive care unit; WBC, white blood cell; χ2, chi-square test

*p < 0.05 was considered to indicate statistical significance

The Lac H0 did not significantly differ between survivors and nonsurvivors. In contrast, the survivors had significantly lower Lac H6 levels, higher ΔLac levels, and higher 6-hour lactate clearance rates than nonsurvivors **(**Table 2**)**. Linear regression analysis revealed no significant differences in lactate levels, duration of mechanical ventilation, or duration of PICU stay between the two groups.

**Table 2:** Distribution of lactate levels between survivors and nonsurvivors

	Overall (*N* = 46)	Survivors (*N* = 21)	Nonsurvivors (*N* = 25)	Test of significance	*P value
Lac H0 (mmol/L), median (IQR)	5.2 (2.7–18)	5.2 (2.7–14)	5.2 (3.1–18)	U = 238	0.59
Lac H6 (mmol/L), median (IQR)	2.2 (0.7–14)	2.1 (0.7–13.5)	2.9 (1.1–14)	*U = 213.5	*0.03
Delta change, (mmol/L) median (IQR)	2.5 (0.3–6)	3.1 (0.5–6)	2.2 (0.3–4.6)	*U = 175.5	*0.04
Lactate clearance median (IQR)	55.44 (3.3 − 78.13)	60 (3.57–78.13)	50.98 (3.3–64.6)	*U = 177.5	*0.04

IQR; interquartile quartile range, Lac H0; lactate levels upon admission, Lac H6; lactate levels 6 h later, Mann‒Whitney U test

*p < 0.05 indicates statistical significance

The PICU mortality rate was significantly related to the lactate clearance rate and lactate levels after 6 h. According to the multivariate logistic regression model, this effect persisted after controlling for confounders (age, gender, and comorbidities). The three different lactate clearance rates (10%, 20%, and 30%) were significantly correlated with higher PICU fatality, and a Lac H6 level ≥ 2 mmol/L was significantly associated with increased PICU mortality **(**Table 3**).**

**Table 3:** Logistic regression analysis to predict PICU mortality by lactate levels

Variables	Unadjusted Odds ratio		Adjusted Odds ratio	
	Odds ratio	95% confidence interval	Odds ratio	95% confidence interval
Lac H6	1.11	1.08–1.9	1.24	1.03–1.57
Lac H6 ≥ 2 mmol/L	2.42	1.41–4.55	2.19	1.01–2.42
LC	1.02	1.01–1.09	1.86	1.56–2.80
LC < 10% (*n* = 6)	2.37	1.08–2.66	1.89	1.35–2.24
LC < 20% (*n* = 11)	2.56	1.26–2.73	1.62	1.33–1.91
LC < 30% (*n* = 14)	2.42	1.12–2.60	1.11	1.02–1.52

Lac H0; lactate levels upon admission, Lac H6; lactate levels 6 h later, LC; lactate clearance, PICU; paediatric intensive care unit

The 6-hour lactate clearance had superior prognostic performance in predicting PICU mortality, with a sensitivity of 62.3% and specificity of 60.4% **(**Table 4**)**.

**Table 4:** Predicting PICU mortality using lactate levels and lactate clearance

	Area under the curve (AUC)	Confidence interval		*P* value	Sensitivity	Specificity	Positive predictive value (PPV)	Negative predictive value (NPV)
LC	0.66	0.50	0.82	*0.048	62.3%	60.4%	50.2%	70.6%
LC < 10% (*n* = 6)	0.55	0.42	0.62		45.6%	62.9%	54.6%	62.4%
LC < 20% (*n* = 11)	0.52	0.04	0.96		52.6%	53.3%	58.6%	67.4%
LC < 30% (*n* = 14)	0.61	0.16	0.84		61.2%	58.4%	54.5%	70.6%
Lac H0	0.45	0.22	0.62	0.59	67.3%	74.5%	60.2%	67%
Lac H6	0.51	0.24	0.58	0.28	57.5%	60.1%	50.6%	68.5%
Lac H6 ≥ 2 mmol/L (*n* = 23)	0.54	0.27	0.73		51.5%	43.6%	59.3%	60.4%

Lac H0; lactate levels upon admission, Lac H6; lactate levels 6 h later, LC; lactate clearance, PICU; paediatric intensive care unit

**Fig. 2:**
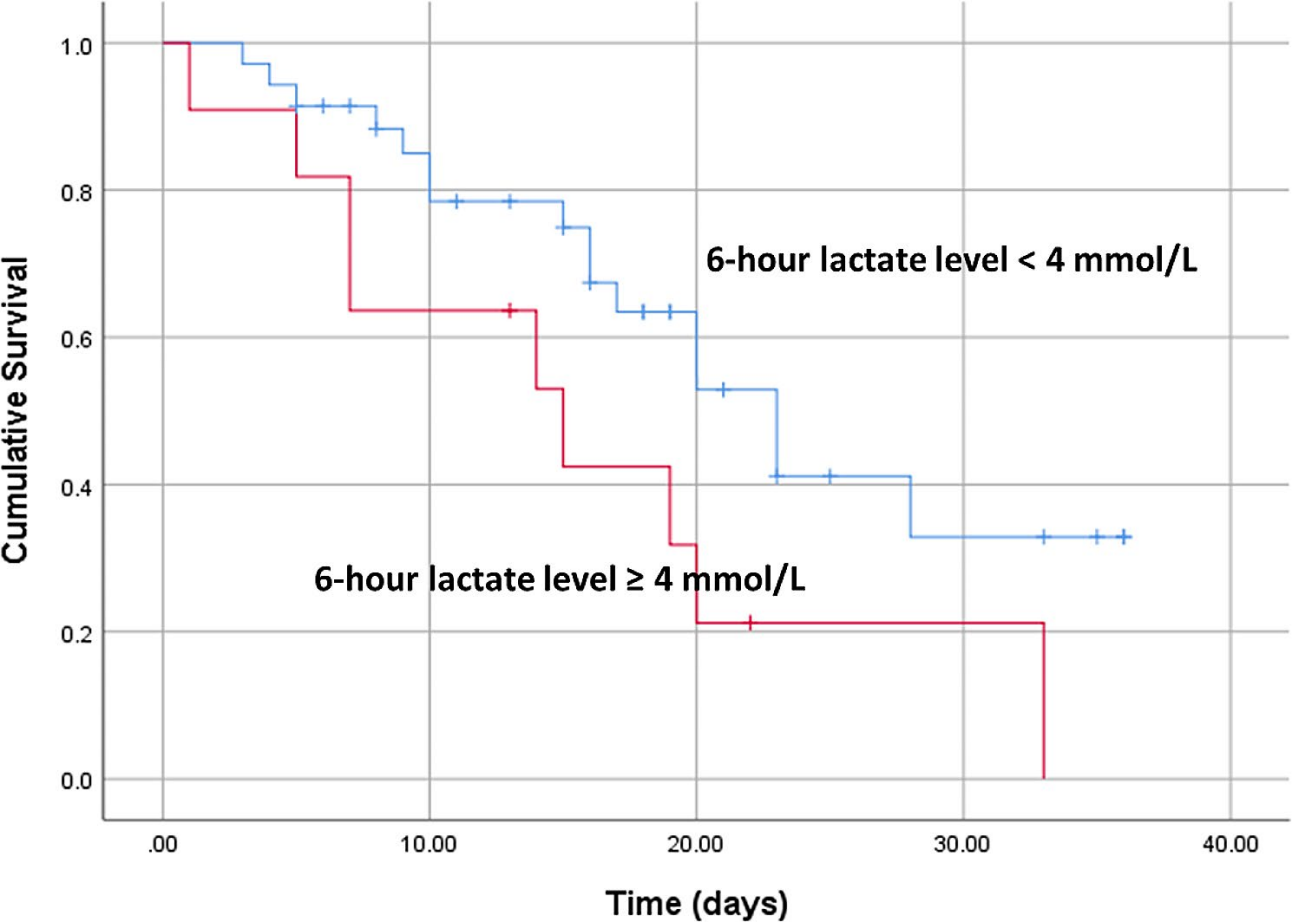
Kaplan–Meier survival curves stratified according to the best lactate cut-off value for six-hour lactate for predicting mortality

**Fig. 3:**
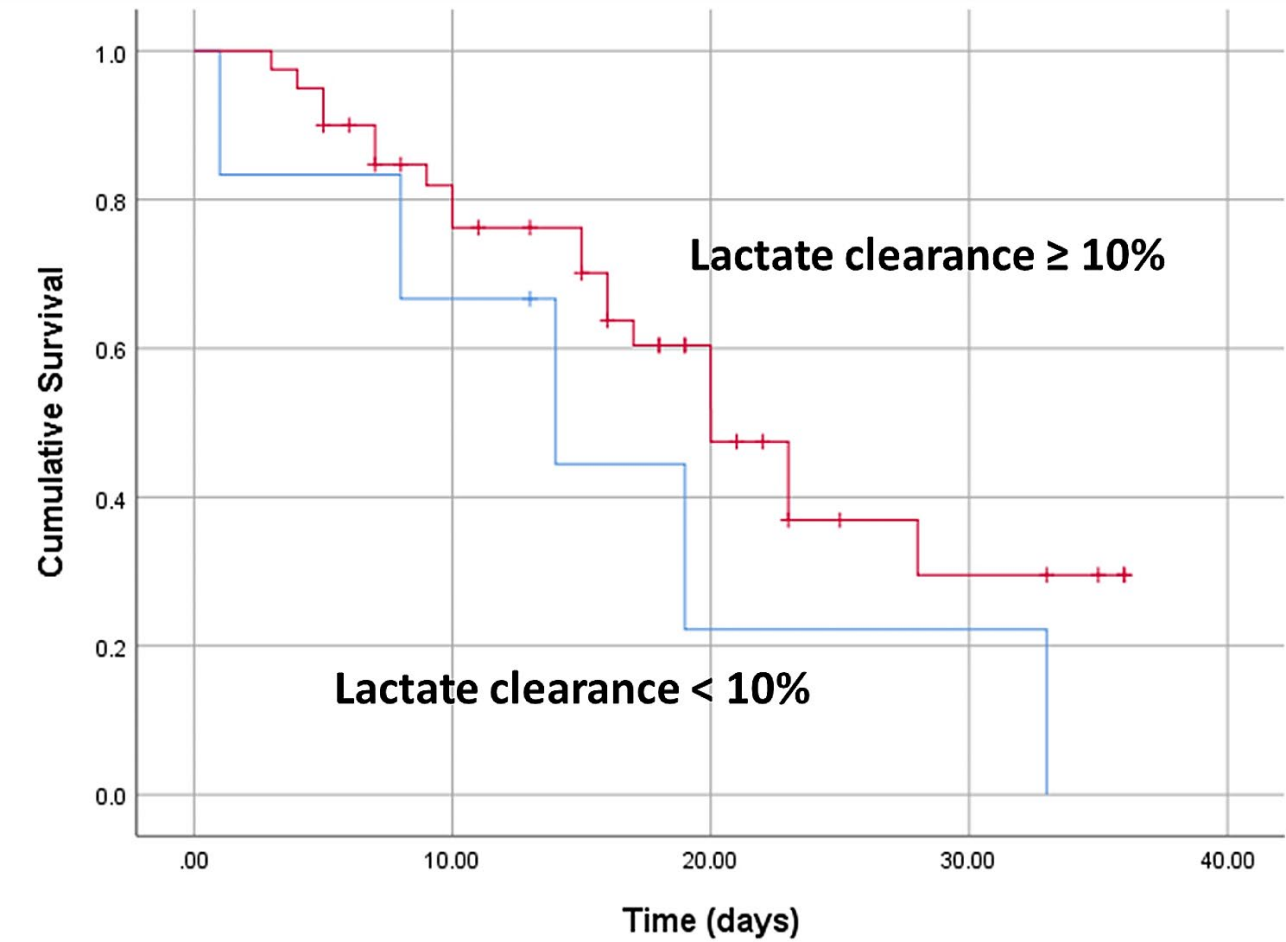
The lactate clearance cut-off value for predicting mortality according to Kaplan–Meier survival curves

Kaplan–Meier survival curve analysis revealed that patients with lower lactate clearance rates and increased Lac H6 levels had significantly higher PICU mortality. The best cut-off values for the lactate clearance rate and Lac H6 for predicting mortality in the PICU were < 10% and ≥ 4 mmol/L, respectively **(**Figs. 2 and 3**)**.

## Discussion

The current study concluded that the survivors had significantly lower Lac H6 levels, greater lactate clearance rates, and greater ΔLac levels than nonsurvivors. In contrast, the Lac H0 did not significantly differ between survivors and nonsurvivors. The best cut-off values for the lactate clearance rate and Lac H6 for the prediction of mortality in the PICU were < 10% and ≥ 4 mmol/L, respectively. Patients with higher Lac H6 levels and lower lactate clearance rates had significantly higher PICU mortality.

Our findings were consistent with those of previous studies evaluating plasma lactate levels in children and adults, as shown in Table 5 [1, 4, 11, 15–19].

**Table 5:** Previous studies evaluating plasma lactate levels in children and adults

Study authors	Type of study	Participants	Methods	Conclusions
Alam and Gupta [11]	Prospective cohort study	A total of 116 children experiencing severe sepsis or septic shock.	Measurements of arterial lactate upon admission (X0) and six hours later (X6). Early mortality was the main outcome.	Lac H6 of 2.65 mmol/L was the best the independent indicator of early mortality risk.
Nazir et al. [15]	Prospective observational study	A total of 112 consecutive children diagnosed with septic shock.	Lactate levels were measured upon PICU admission and at 6-, 12-, and 24-hours postadmission.	The 6-hour lactate clearance had a sensitivity and specificity of 0.948 and 0.571, respectively, at a 10% cut-off in predicting mortality.
Kim et al. [16]	Retrospective observational study	Sixty-five Korean paediatric patients with septic shock.	After admission, consecutive arterial lactate levels were measured one time, and then every six hours for a total of twenty-four hours. The total AUC of the consecutive lactate levels recorded over the 24 h after admission was used to define the lactate area.	Initial lactate levels were significantly associated with death. Patients with lactate levels > 5 mmol/L had higher mortality rates. Lactate clearance is more beneficial for predicting outcomes than a single baseline lactate level. Lactate levels increased and remained high in nonsurvivors but not in those who survived.
Moustafa et al. [17]	Prospective observational study	Seventy-six PICU patients.	Arterial lactate levels were measured at H0 and H6. Patients were separated into two different categories. Category A had a 6-hour lactate clearance > zero (improved lactate), while Category B had a 6-hour lactate clearance ≤ zero (escalated lactate).	The arterial lactate level at H0 was a weak indicator of death. However, the 6-hour lactate clearance was a strong predictor, with a clearance of 0% indicating fatality and appropriate level of sensitivity and specificity (70.8% and 90.4%, respectively).
Kumar and Kumar [18]	Prospective, observational study.	A total of 140 PICU patients.	A positive score indicates a reduction in lactate, whereas a negative score indicates a rise in lactate after six hours of management.	The AUC for 6-hour lactate clearance was 0.823.
Munde et al. [1]	Pilot study	A convenience sample of 45 consecutive patients in the PICU. .	Lactate levels were measured upon admission and after six hours, and clearance was determined. A positive score shows lactate clearance, whereas a negative score indicates an increase in lactate levels.	At 6 h, a lactate clearance of less than 30% predicted death with a sensitivity of 75% and a specificity of 97%.
Marty et al. [19]	Prospective, observational case series of adult patients in a surgical ICU.	A total of 94 adult patients with severe sepsis or septic shock.	Lactate levels were assessed at 6, 12, and 24 h after admission. A positive score represents a reduction in lactate rate.	A lactate clearance at 6 h predicted mortality with 63.46% sensitivity and 56.1% specificity.
Choudhary et al. [4]	Prospective observational study	A total of 148 Pediatric septic patients.	Sequential lactate measurements were assessed upon admission to the PICU as well as twenty-four and forty-eight hours later. The main outcome metric was survival or death after the hospital stay.	An initial lactate value of 4 mmol/L was an acceptable predictor of death (sensitivity = 55%, and specificity = 82%), and the best cut-off for lactate clearance (AUC = 0.755) was found to be less than 10%.

The conclusions of the present study are also consistent with those of other studies showing that initial lactate levels have poor sensitivity or specificity for predicting mortality [20–22]. To anticipate the consequences in critically ill children, sequential lactate measurements are essential instead of single measurements. Lactate clearance is considered a promising biomarker of microcirculation because it is strongly related to capillary perfusion. Septic shock is characterised by microcirculatory disruption, decreased oxygen supply to tissues and organs, and impaired organ function. Multiple-organ dysfunction syndrome, recognised as an important predictor of death, arises from persistently inadequate blood flow to vital organs [20–22].

Notably, the AUC for lactate clearance was low in our study. This could be attributed to the variety of confounders, limited total number of patients, insufficient sample size, clinical condition of our patients, and increased mortality rates.

Chertoff et al. [23] reported that within the first 8 h of hospital admission, patients with lactate clearance greater than 20% had a 22% reduced risk of mortality compared to those with clearance less than 20%. Additionally, an 11% reduction in mortality was observed for each 10% increase in lactate clearance. Although previous studies have differed in terms of geographical area, sample size, patient characteristics, and testing methods, there is increasing evidence supporting the utility of lactate measurements, such as lactate clearance, in mortality risk stratification, suggesting that this approach would be helpful in clinical practice.

Compared to that of Lac H0, the 6-hour lactate clearance more precisely reflects a child’s clinical status because the response to resuscitation throughout the first 6-hour time frame is considered. This finding reinforces the notion that lactate levels trending over time are better than lactate levels at predicting in-hospital mortality. Lac H6 levels returned to normal after a good response to treatment during the initial six hours. On the other hand, a poor response to treatment results in a higher Lac H6 with reduced lactate clearance, likely leading to adverse consequences and necessitating a greater level of care. Clinicians benefit from this approach because it allows them to identify high-priority children quickly.

In contrast to our findings, Nichol et al. [24] reported that low levels of lactate were correlated with a greater likelihood of death in adult patients. In addition, Wacharasint et al. [25] observed a greater fatality rate in adult septic shock patients with lower lactate levels. In their study of critically ill children, Koliski et al. [26] reported that blood lactate levels (Lac H0 and Lac H12) were ineffective at predicting patient mortality risk. Gupta A et al. [27] reported that the mean lactate level during the initial 6 h of hospitalisation in critically ill children in India is a more reliable indicator of prognosis than the lactate clearance during the exact duration of hospitalisation.

Lactate levels may be normal in some patients with sepsis, and lactate clearance testing is ineffective in these patients. Many lactate measurement confounders exist, such as the administration of Ringer’s lactate as an emergency resuscitation fluid in patients with septic shock, which may delay lactate clearance, the presence of liver disease in these patients, which may delay lactate clearance monitoring, the administration of exogenous lactate producers such as adrenaline, which promotes pyruvate-to-lactate conversion in the lungs and increases lactate release into the systemic circulation, and the need for a large quantity of transfused red blood cells [28, 29]. Therefore, alternative variables should be used to predict the prognosis of septic shock patients.

The strength of this study is the choice of a 6-hour time window to evaluate the impact of resuscitation on lactate levels. Since we measured lactate levels early and after 6 h, our study suggests that high levels of lactate in patients with severe sepsis are associated with mortality. This study was performed at a tertiary hospital that serves a large general population, which improved the quality of the study.

Therefore, significant constraints must be considered when evaluating our results. The small sample size, observational single-center study design, and inability to rule out the effects of drugs such as epinephrine are the limitations of this study. Furthermore, the high fatality rate (54% of the recruited sample) may limit the generalizability of our findings. Additional multicenter clinical trials are needed to define the lactate clearance threshold for directed treatment in patients with severe sepsis and septic shock.

## Conclusion

This study highlights the significance of lactate level trends over time in predicting mortality in children with severe sepsis or septic shock. Elevated plasma lactate levels and reduced lactate clearance after 6 h of hospitalisation are associated with a higher mortality rate, highlighting the importance of these variables as independent, readily available, and crucial factors for assessing mortality risk.

## Data Availability

The datasets generated and/or analysed during the current study are not publicly available but are available from the corresponding author upon reasonable request.
